# Abrogation of epithelial BMP2 and BMP4 causes Amelogenesis Imperfecta by reducing MMP20 and KLK4 expression

**DOI:** 10.1038/srep25364

**Published:** 2016-05-05

**Authors:** Xiaohua Xie, Chao Liu, Hua Zhang, Priyam H. Jani, Yongbo Lu, Xiaofang Wang, Bin Zhang, Chunlin Qin

**Affiliations:** 1 Department of Stomatology, and Institute of Hard Tissue Development and Regeneration, the 2^nd^ Affiliated Hospital of Harbin Medical University, Harbin, 150086, China; 2 Department of Biomedical Sciences and Center for Craniofacial Research and Diagnosis, Texas A&M University Baylor College of Dentistry, Dallas, Texas, 75246, USA; 3 Department of Oral Biology, College of Stomatology, Dalian Medical University, Dalian, 116044, China; 4 Heilongjiang Academy of Medical Sciences, Harbin, 150001, China

## Abstract

Amelogenesis Imperfecta (AI) can be caused by the deficiencies of enamel matrix proteins, molecules responsible for the transportation and secretion of enamel matrix components, and proteases processing enamel matrix proteins. In the present study, we discovered the double deletion of bone morphogenetic protein 2 (*Bmp2*) and bone morphogenetic protein 4 (*Bmp4*) in the dental epithelium by *K14-cre* resulted in hypoplastic enamel and reduced density in X-ray radiography as well as shortened enamel rods under scanning electron microscopy. Such enamel phenotype was consistent with the diagnosis of hypoplastic amelogenesis imperfecta. Histological and molecular analyses revealed that the removal of matrix proteins in the mutant enamel was drastically delayed, which was coincided with the greatly reduced expression of matrix metalloproteinase 20 (MMP20) and kallikrein 4 (KLK4). Although the expression of multiple enamel matrix proteins was down-regulated in the mutant ameloblasts, the cleavage of ameloblastin was drastically impaired. Therefore, we attributed the AI primarily to the reduction of MMP20 and KLK4. Further investigation found that BMP/Smad4 signaling pathway was down-regulated in the *K14-cre;Bmp2*^*f*/*f*^*;Bmp4*^*f*/*f*^ameloblasts, suggesting that the reduced *MMP20* and *KLK4* expression may be due to the attenuated epithelial BMP/Smad4 signaling.

Tooth enamel, the hardest tissue in the human body, contains inorganic and organic components. The inorganic components are made of highly organized hydroxyapatite (HAP) crystals while the organic components consist of matrix proteins produced by ameloblasts, including amelogenin (AMEL), ameloblastin (AMBN), enamelin (ENAM), amelotin (AMTN) and odontogenic ameloblast-associated protein (ODAM). Mutations in, or deletions of, the genes encoding AMEL, AMBN and ENAM have been reported to cause Amelogenesis Imperfecta (AI) in humans and mice^1^,^2^,^3^,^4^,^5^,^6^,^7^,^8^. Additionally, ameloblasts also secrete matrix metalloproteinase 20 (MMP20, also called “enamelysin”) at the secretory stage and “kallikrein 4” (KLK4) at the maturation stage to cleave and remove these matrix proteins before the enamel matrix is fully mineralized^9^. For example, current opinion thinks that the full-length ENAM of 186 kDa is cleaved into a 32 kDa fragment by MMP20 during secretory stage, which serves as nucleators for the formation of HAP crystals, while during the maturation stage, these 32 KDa fragments of ENAM are further degraded by KLK4 into smaller products^10^. Inactivation of *MMP20* and/or *KLK4* leads to hypoplastic and hypomineralized AI in both human subjects and mice^7^,^11^,^12^,^13^,^14^,^15^. Further analyses demonstrated that the cleavage of AMEL was impaired in the *MMP20* deficient mice^11^, and hypomature AI in the *KLK4* knock-out/*LacZ* knock-in mice was characterized by significantly retained ENAM in the mature enamel matrix^15^. All these results demonstrate that the processing of enamel matrix proteins by MMP20 and KLK4 is essential for the normal process of amelogenesis.

Recently, mutations of *Fam20a*, *Fam83h* and *LAMB3*, which may be essential for posttranslational modification, transportation and secretion of enamel matrix proteins, have been identified in human AI^16^,^17^,^18^, indicating that the molecular basis of human AI is more than enamel matrix proteins and proteinases. Based on these findings, we speculate that the functional loss of molecules regulating the expression of the secretory matrix proteins and proteinases during amelogenesis can be also associated with AI. As one of the most important classes of growth factors, the functions of bone morphogenetic proteins (BMPs) have been intensively studied in the early-stage tooth development^19^,^20^,^21^,^22^,^23^, but the exact roles of BMPs in the differentiation and maturation of ameloblasts remain to be elucidated. Recently, it is reported that the deletion of *Bmp2* from differentiating odontoblasts results in hypolastic enamel in mice with a decreased level of MMP20^24^. Since *Bmp2* and *Bmp4* are transcribed in the ameloblasts at the secretory stage^19^,^20^, we postulate that the *Bmp2* and *Bmp4* expressed in the secretory ameloblasts may control the production and processing of the enamel matrix proteins. To test this hypothesis, we specifically ablated *Bmp2* and *Bmp4* from mouse epithelium by breeding the *Bmp2-* and *Bmp4-*floxed mice with *K14-cre* mice and analyzed the enamel in the double *Bmp2-* and *Bmp4*-knockout mice.

## Results

### Amelogenesis Imperfecta in *K14-cre;Bmp2* ^*f*/*f*^ *;Bmp4* ^*f*/*f*^mice

**Figure 1:**
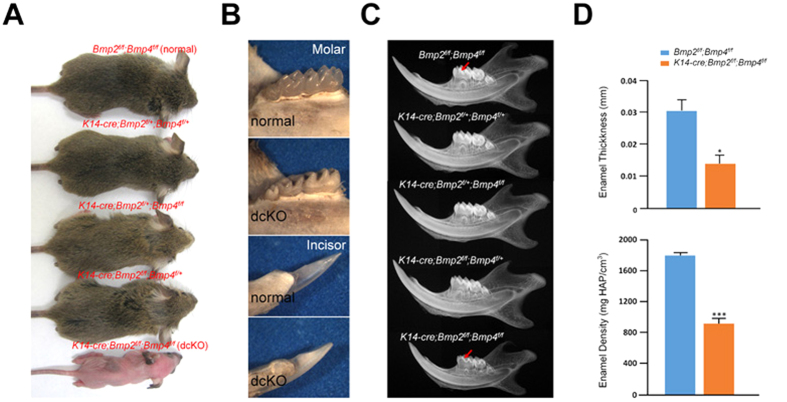
Amelogenesis Imperfecta in *K14-cre;Bmp2*^*f*/*f*^*;Bmp4*^*f*/*f*^mouse. (**A**) At postnatal 3 weeks (P3w), there was no significant difference by their appearance among the *K14-cre;Bmp2*^*f*/+^*;Bmp4*^*f*/+^*, K14-cre;Bmp2*^*f*/*f*^*;Bmp4*^*f*/+^, *K14-cre;Bmp2*^*f*/+^*;Bmp4*^*f*/*f*^ mice and *Bmp2*^*f*/*f*^*;Bmp4*^*f*/*f*^(normal) littermates. However, the *K14-cre;Bmp2*^*f*/*f*^*;Bmp4*^*f*/*f*^(dcKO) mouse had hairless skin. (**B**) At P4w, the molar and incisor in the normal mice were well mineralized and semitransparent, but the teeth in the dcKO mice exhibited a chalk-like appearance. (**C**) Plain X-ray of the mandibles from P3w mice. The dcKO mice had thinner enamel (arrows) compared with the normal littermates. (**D**) At P3w, both the enamel thickness and mineral density of the first lower molar, as assessed by micro-CT, had dramatically decreased in dcKO mice compared to those of the normal controls. Error bar represents SD. *P < 0.05; *** *P* < 0.001.

At postnatal three weeks (P3w), the *K14-cre;Bmp2*^*f*/+^*;Bmp4*^*f*/+^*, K14-cre;Bmp2*^*f*/*f*^*;Bmp4*^*f*/+^ and *K14-cre;Bmp2*^*f*/+^*;Bmp4*^*f*/*f*^ mice could not be distinguished from their *Bmp2*^*f*/*f*^*;Bmp4*^*f*/*f*^(normal) littermates by their appearance (Fig. 1A). In contrast, the *K14-cre;Bmp2*^*f*/*f*^*;Bmp4*^*f*/*f*^(dcKO) mice exhibited hairless skin (Fig. 1A), which was likely due to the regression of hair follicles^29^. Both the incisors and molars in the dcKO mice had a chalk-like appearance with lower transparency and poor reflection under bright light, which was obviously different from normal teeth (Fig. 1B). Plain X-ray images indicated that the enamel thickness was significantly reduced in the dcKO mice, but there were no evident changes in other groups of mice compared with the normal controls (Fig. 1C). Consistent with the plain X–ray images, the statistical analysis of the micro–CT scans indicated that both the thickness and the mineral density of enamel in the dcKO mice were significantly reduced compared to those of the normal controls (Fig. 1D). These results indicated that the double deletion of *Bmp2* and *Bmp4* from the dental epithelium caused amelogenesis imperfecta (AI) in these mice.

### Impaired removal of enamel matrix in the *K14-cre;Bmp2* ^*f*/*f*^ *;Bmp4* ^*f*/*f*^ mice

**Figure 2:**
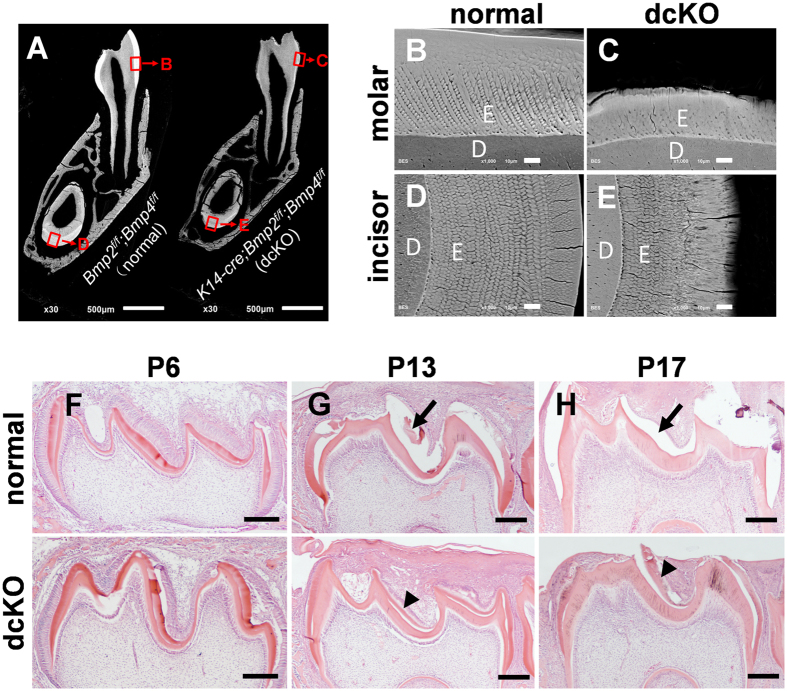
Hypoplastic enamel and retained enamel matrix in dcKO mouse. (**A**) SEM analyses of mesial roots from P4w dcKO mice and normal littermates. The enamel of both the molar and incisor was obviously thinner in the dcKO mice, compared with normal teeth. (**B–E**) Higher magnification views of the boxed areas in (**A**). In the dcKO mice, the teeth had the shortened enamel rods and rough enamel surfaces (**C,E**), compared with normal littermates (**B**,**D**). The boundary between the enamel rods was blurry (**C**). (**F–H**) H&E-stained sections of mandible 1^st^ molars. At P6, there was no difference in continuity or thickness of the enamel layer between the normal and dcKO mice (**F**). In the normal molar, most of the enamel matrix in the cusp region was lost at P13 ((**G**) arrow), and all the enamel matrix was gone in the demineralized paraffin sections at P17 ((**H)**, arrow). However, the enamel matrix was still present in the molars of the dcKO mice at P13 and P17 ((**G,H**), arrowheads). (**D**) dentin; (**E**) enamel. Scale bars: 500 μm in A; 10 μm in (**B–E**) and 200 μm in (**F–H**).

SEM analyses further confirmed the Amelogenesis Imperfecta phenotypes in the dcKO mice. The dentin thickness of the postnatal 4-week-old (P4w) dcKO mice was comparable to that of the control mice, while the enamel in the dcKO mice was much thinner than normal (Fig. 2A). The hypoplastic enamel of dcKO mice exhibited not only shortened enamel rods in deeper layer, but also a reduced thickness of prismatic layer (Fig. 2B–E). Moreover, the boundary between the inter rods and the enamel rods in the molars of the dcKO mice became blurry (Fig. 2C). H&E staining revealed how the AI developed in the dcKO enamel. At postnatal day 6 (P6), the 1^st^ molar crowns of both the dcKO and the normal mice were covered by a continuous enamel layer in the demineralized paraffin sections (Fig. 2F). At postnatal day 13 (P13), the enamel matrix was absent in the cusp region, but still retained in the crown-root junction of normal molar. In contrast, a considerable amount of enamel matrix was still retained in the cusp region of dcKO samples (Fig. 2G), suggesting a delay in the removal of matrix proteins and a defect in mineralization during amelogenesis in the dcKO mice. At P17, this matrix-retaining defect in the dcKO mice became more remarkable compared to the normal mice: the enamel matrix existed in the molar of the dcKO mice, but completely disappeared in the demineralized normal molar (Fig. 2H). These analyses indicated that the proteolytic degradation of enamel matrix proteins in the dcKO mice was impaired and subsequently, resulted in the retention of the organic enamel matrix in the demineralized paraffin sections of molars at P17 day.

### Reduced expression levels of MMP20 and KLK4 in *K14–cre;Bmp2* ^*f*/*f*^ *;Bmp4* ^*f*/*f*^ ameloblasts

**Figure 3:**
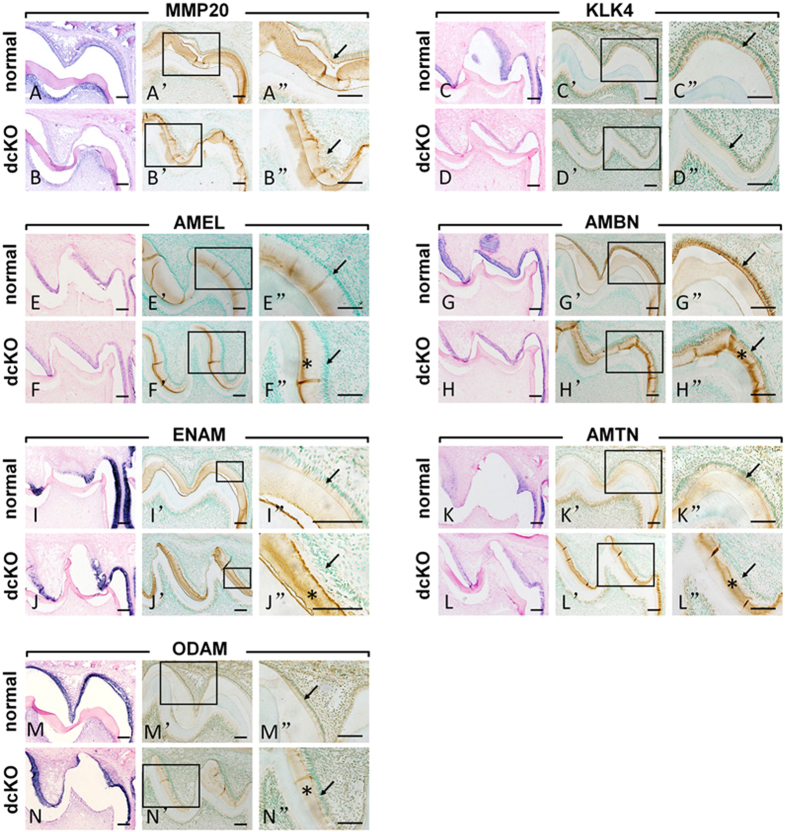
*In situ* hybridization and immunohistochemical analyses of enamel matrix proteases and proteins in the 1^st^ molar of the P10 mandible. (**A**–**N**) The mRNA levels of MMP20 (**A**,**B**), KLK4 (**C**,**D**), AMBN (**G**,**H**) and ENAM (**I,J**) were lower in the dcKO ameloblasts than in the normal controls. There were no obvious differences in the transcription levels of AMEL (**F,F’**), AMTN (**K,L**) and ODAM (**M,N**) in the ameloblasts between the normal and dcKO mice. (**A’–N’**) Immunohistochemistry of MMP20 (**A’,B’**), KLK4 (**C’,D’**), AMEL (**E’,F’**), AMBN (**G’,H’**), ENAM (**I’,J’**), AMTN (**K’,L’**) and ODAM (**M’,N’**) in the molars of the dcKO and normal mice. (**A”–N”**) Higher magnification views of the boxed areas in (**A’–N’**) (arrows point to ameloblasts). The cytoplasm of the ameloblasts in the 1^st^ molars of the dcKO mice appeared to have reduced levels of MMP20 (**B’,B”**), KLK4 (**D’,D”**), AMEL (**F’,F**), AMBN (**H’,H”**), ENAM (**J’,J”**), AMTN (**L’,L”**) and ODAM (**N’,N”**) compared with those of the normal ameloblasts (**A’,A”; C’,C”; E’,E”; G’,G”; I’,I”; K’,K” and M’,M”**). The immunostaining of MMP20 (**B”**) and KLK4 (**D”**) in the enamel matrix of the dcKO mice was weaker than in the normal enamel (**A”,C”**). Intensive and uneven deposition of AMEL, AMBN, ENAM, AMTN, ODAM was seen in the deep layer of enamel in the dcKO mice (asterisks in **F”**,**H”**,**J”**,**L”** and **N”** indicate the strong staining of these proteins in certain areas of the enamel matrix). Scale bars: 200 μm.

*In situ* hybridization analyses revealed that at P10, the transcription level of *MMP20* and *KLK4* was lower in the ameloblasts of the dcKO mice than that in the normal mice (Fig. 3A–D). Consistently, immunohistochemistry revealed reduced levels of MMP20 and KLK4 proteins in the cytoplasm of the dcKO ameloblasts and in the retained matrix of the enamel at P10 (Fig. 3A’”). The mRNA levels of AMEL, AMTN and ODAM did not seem to be affected by the double deletion of *Bmp2* and *Bmp4* (Fig. 3E,F,K–N). In contrast, the transcript levels of AMBN and ENAM were lower in the ameloblasts of the dcKO mice than those in the normal mice (Fig. 3G–J). Immunohistochemistry analyses showed that while AMEL protein level in the cytoplasm of the ameloblasts had no obvious difference between the dcKO and control mice (Fig. 3E,E’), the protein levels of AMBN, ENAM, AMTN and ODAM were lower in the cytoplasm of the ameloblasts of P10 dcKO molars than in the normal molar (Fig. 3G’–N’). On the other hand, AMEL, AMBN, ENAM, AMTN and ODAM showed stronger immunoreactivities in the enamel matrix of dcKO mice than those in the normal counterparts (Fig. 3E”–N”). Additionally, all the enamel matrix proteins distributed unevenly, particularly highly concentrated in the deep layer of dcKO enamel (asterisks in Fig. 3F””). Such deposition pattern strongly suggested that the down-regulation of MMP20 and KLK4 impaired the proteolytic processing of the enamel matrix proteins in the dcKO mice.

### Impaired cleavage of AMBN in the *K14-cre;Bmp2* ^*f*/*f*^ *;Bmp4* ^*f*/*f*^enamel matrix

**Figure 4:**
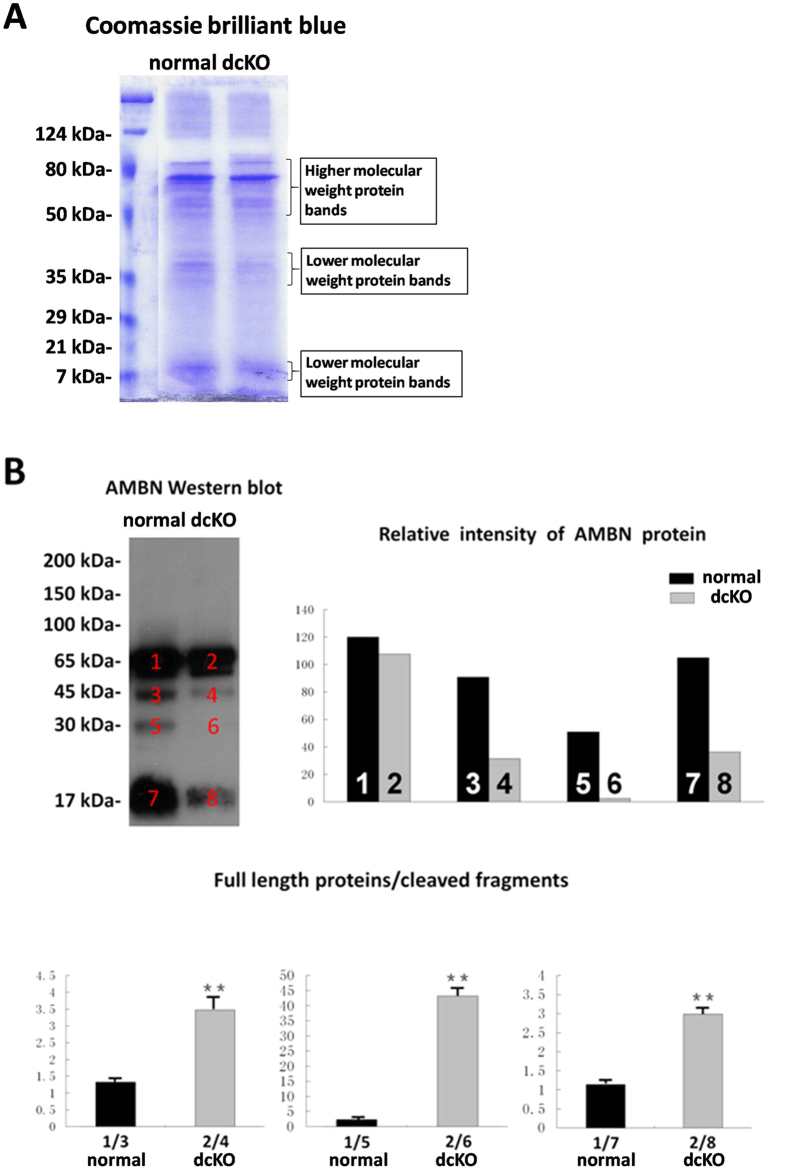
Coomassie Brillaint Blue staining of the total protein extract and anti-AMBN Western blotting analysis. (**A**) Coomassie Brillaint Blue staining showed four clusters of protein bands. The most prominent cluster of protein bands migrated between 80 and 50 kDa; the major protein band at approximately 70 kDa was likely to be full-length AMEL. The cluster of protein bands just above the 35 kDa marker and that around 7 kDa were likely to be the cleaved products of enamel matrix proteins. (**B**) The anti-AMBN Western blotting analysis revealed that the ratio of full-length AMBN to its cleaved fragments was significant greater in the dcKO mice than in the normal mice, indicating a poor degradation of this enamel matrix protein in the dcKO mice. Full-length AMBN was at about 65  kDa, and the protein bands present between 17 and 45 kDa were AMBN fragments. *P < 0.05; **P < 0.01.

To analyze the proteolytic processing of the enamel matrix proteins in dcKO and normal mice, total protein was extracted from the enamel-forming epithelial organ and enamel matrix of 1^st^ molars at 5 days after birth. Coomassie Brilliant Blue staining (Fig. 4A) showed that the protein bands of lower molecular weight in the enamel of dcKO mice were remarkably weaker than the normal control mice, while the protein bands of higher molecular weight in the former mice were similar to the latter. These observations indicate that overall, the enamel matrix of the dcKO mice may have a reduction in the amounts of cleaved products for certain enamel matrix proteins, in relativity to the full-length forms of these molecules. To confirm the cleavage impairment, anti-AMBN Western blotting analysis was performed. When equal amounts of proteins were loaded, the intensities of protein bands representing full-length AMBN (65 kDa) showed no significant difference between dcKO and normal enamel (Fig. 4B). However, the protein bands for the cleaved products of AMBN (45, 30 and 17 kDa) in dcKO molars displayed the much weaker intensities (Fig. 4B), suggesting that the cleavage of AMBN in dcKO enamel was impaired.

### Attenuated BMP/Samd4 signaling pathway in *K14-cre;Bmp2* ^*f*/*f*^ *;Bmp4* ^*f*/*f*^ ameloblasts

**Figure 5:**
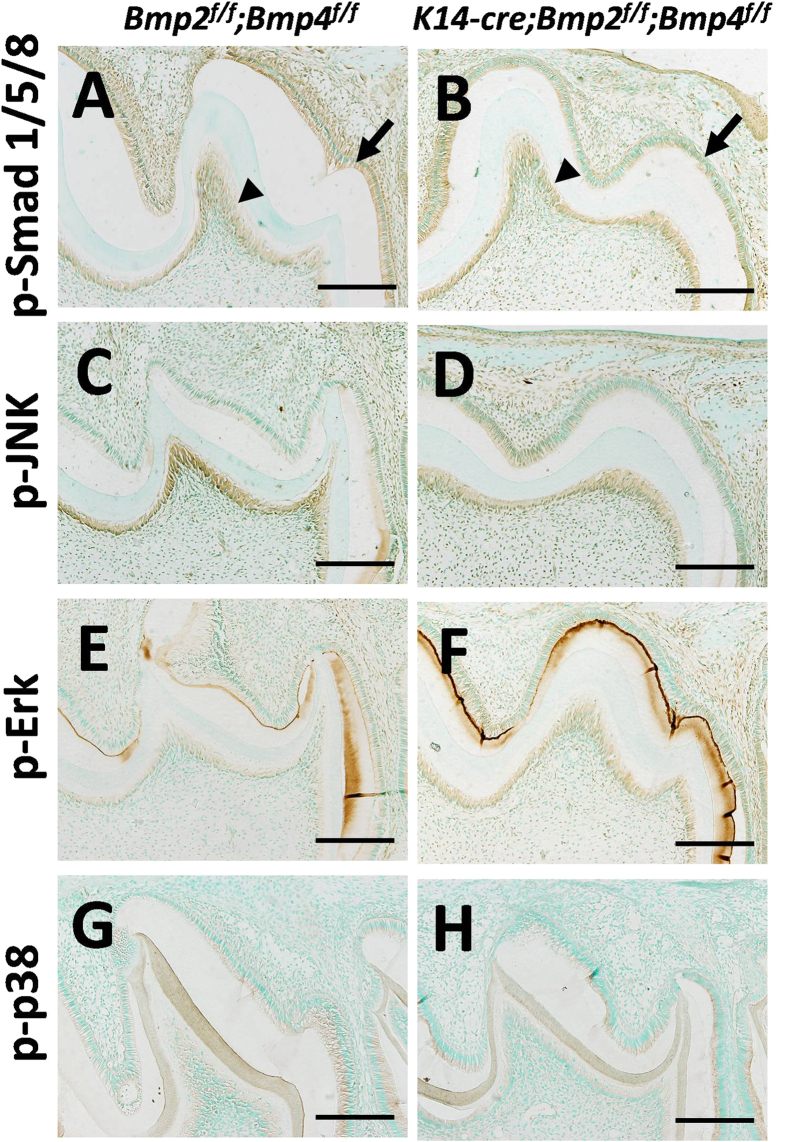
Immunohistochemistry of p-Smad 1, 5 and 8, p-JNK, p-Erk and p-p38 in the 1^st^ molar of mandibles in P9 mice. (**A,B**) The expression of p-Samd1, 5 and 8 was significantly down-regulated in the dcKO ameloblasts (**A**, arrow), but not in the odontoblasts (**A**, arrowhead), compared with normal control (**B**, arrow and arrowhead). (**C–H**) There was no difference in the levels of p-JNK (**C,D**), p-Erk (**E,F**) and p-p38 (**G,H**) between the dcKO and normal controls. Scale bars: 200 μm.

**Figure 6:**
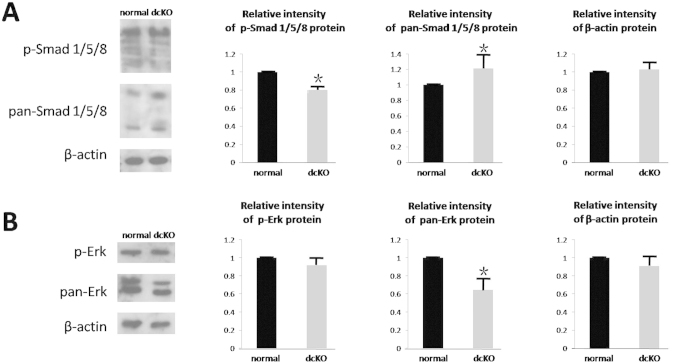
Western blotting analysis for the levels of BMP/Smad4 and BMP/Erk signaling in the enamel organs of the 1^st^ mandibular molar of P9 mice. (**A**) Compared with the normal control, the amount of pan-Smad 1/5/8 in dcKO elevated remarkably, while the p-Smad 1/5/8 significantly decreased. The ratio of p-Smad 1/5/8 to pan-Smad 1/5/8 dropped from 1 in normal control to 0.593 in dcKO. (**B**) Reversely, the amount of pan-Erk decreased dramatically in dcKO, while the p-Erk showed no difference when compared with the normal counterparts. Therefore, the ratio of p-Erk to pan-Erk increased from 1 in the control to 1.58 in dcKO. All ratios have been normalized by the relative amount of β-actin. *P < 0.05.

BMP2 and BMP4 activate Smad 1, 5 and 8, which in turn form a complex with phosphorylated Smad4 to activate down-stream targets. Besides the BMP/Smad4 signaling pathway, BMP2 and BMP4 can also activate the Erk/Mek, p38/MAPK and JNK signaling pathways to regulate the relevant biochemical processes and gene expression^30^. Immunohistochemistry analyses revealed that the level of phosphorylated Smad 1, 5 and 8 (p-Smad 1, 5 and 8) was obviously down-regulated in the ameloblasts of the dcKO mice at P9, but not in the odontoblasts (Fig. 5A,B). In contrast, the JNK, Erk and p-38 signaling pathways showed no differences between dcKO and normal controls (Fig. 5C–H). To confirm the immunohistochemistry findings, Western blotting bands of Smad 1/5/8 and Erk in P9 enamel organs were quantified. Consistent with the immunohistochemistry results, both the amount of p-Smad 1/5/8 and the ratio of p-Smad 1/5/8 to pan-Smad 1/5/8 were significantly decreased in the dcKO enamel organs (Fig. 6A). On the other hand, although the p-Erk level in dcKO was comparable to that of control, the amount of pan-Erk in dcKO decreased dramatically compared with pan-Erk in normal control (Fig. 6B). Therefore, we concluded that the epithelial BMP2 and BMP4 activated canonical BMP/Smad4 pathway in the secretory and differentiating ameloblasts. Combined with reduced expression of *MMP20* and *KLK4* in the dcKO ameloblasts, these results suggested that the BMP2 and BMP4 in secretory and differentiating ameloblasts may activate *MMP20* and *KLK4* expression through the canonical BMP/Smad4 pathway.

## Discussion

*Bmp2* and *Bmp4* are expressed in the enamel knots during tooth morphogenesis and in pre-ameloblasts during enamel maturation^19^,^20^. In the present study, we employed *K14-cre* mice, which express the cre-recombinase in the dental epithelium around E10.5^31^,^32^,^33^, to inactivate both *Bmp2* and *Bmp4* in the epithelium. The double deletion of *Bmp2* and *Bmp4* in the epithelium at this time point led to hypoplastic enamel without a deficiency in the tooth morphology. The unaffected tooth morphology can be interpreted as the functional redundancy among *Bmp2*, *Bmp4* and other *Bmps*, especially *Bmp7* which has been shown to play an important role in tooth morphogenesis^19^,^20^.

The remarkable enamel defects observed in the double *Bmp2-* and *Bmp4-*knockout mice indicated the indispensable role of these two growth factors in amelogenesis. At the secretory stage, the dcKO mice exhibited polarized, high columnar ameloblasts, and produced an enamel layer, the thickness of which was comparable to the normal controls, indicating that the expression of *Bmp2* and *Bmp4* in the dental epithelium after E10.5 was not essential to the differentiation of ameloblasts and the secretion of enamel matrix proteins. However, the retention of the enamel matrix at the maturation stage, the shortened enamel rods and reduced thickness of mineralized enamel in the dcKO mice suggested impaired processing (removal) of enamel matrix proteins in the double *Bmp2-* and *Bmp4-*deficient mice. Previous studies demonstrated that the hypoplastic enamel in amelogenesis imperfecta could result from a failure to process enamel matrix proteins by the ameloblast-synthesized proteinases, as well as from the lack of a sufficient production of these matrix molecules^1^,^2^,^3^,^4^,^5^,^6^,^7^,^8^,^10^,^11^,^12^,^14^,^34^. In this study, the comparable thickness at the secretory stage and retained enamel matrix of the dcKO mouse molars suggested that the secretory dcKO ameloblasts produced sufficient enamel matrix proteins to serve as the presumptive scaffold required for enamel mineralization. Therefore, the hypoplastic enamel in the dcKO mice was most likely attributed to the dramatic down-regulation of MMP20 and KLK4, which led to the insufficient removal of the enamel matrix. These findings suggest that BMP2 and BMP4 in the dental epithelium play an essential role in regulating the expression of MMP20 and KLK4 in this tissue after E10.5.

The retained enamel matrix of the dcKO mice showed stronger but uneven depositions of the five matrix proteins: AMEL, AMBN, ENAM, AMTN and ODAM than in the normal mice. These observations lent further support to our belief that the reduced proteolytic activity associated with the down-regulation of MMP20 and KLK4 was likely responsible for the development of hypoplastic AI during the maturation stage. Furthermore, since the immnuohistochemical results showed little difference in AMEL level in the cytoplasm of ameloblasts between the dcKO and normal mice, and Coomassie Brilliant Blue staining indicated that the full length AMEL may not be significantly affected in the dcKO molars, we chose AMBN, the mutations of which were associated with AI, for Western blotting analysis.

Coomassie Brilliant Blue staining showed that overall, the protein bands of lower molecular weight in the enamel of dcKO mice were remarkably weaker than in the normal mice. The anti-AMBN Western blotting analysis showed that the enamel of dcKO had much less AMBN fragments in relativity to its full-length form than the normal mice. These observations provide further support that the cleavage of certain enamel matrix proteins in the dcKO mice was defective due to the reduced levels of MMP20 and KLK4. MMP20 cleaves amelogenin, ameloblastin and amelotin at the secretory stage, and KLK4 performed a similar proteolytic function at the following stages of transition and maturation^9^. The hypoplastic enamel in the MMP20-deficient mice delaminated from the dentin had an altered rod pattern because of the impaired processing of the enamel matrix^1^. Although the delaminated enamel was not detected in the teeth of our dcKO mice, the ultramicroscopic observation revealed that these mice have shortened enamel rods and thinner prismatic enamel, which are identical to those observed in the enamel of MMP20-deficient mice. Since the transgenic MMP20 allele rescued the AI in the MMP20-deficient mice^35^, further studies are warranted to see if expressing this transgene can rescue or improve the hypoplastic enamel in the dcKO mice.

A recent study reported that *Bmp2* deletion in the dental mesenchyme mediated by the *Osx-cre* also resulted in dysplastic dentin, along with the reduction in the volume and density of enamel^24^. The *Osx-cre;Bmp2*^*f*/*f*^mice showed a reduced expression of ENAM, MMP20 and KLK4 in the ameloblasts and MMP20 in the *Bmp2*-difficient odontoblasts^24^. Compared to the hypoplastic enamel and dysplastic dentin in *Osx-cre;Bmp2*^*f*/*f*^ mice, our dcKO mice manifested only enamel defects without dentin abnormality. Such discrepancy may result from the indispensable role of mesenchyme BMP2 in the reciprocal interactions between dental mesenchyme and epithelium, and the functional redundancy between epithelial BMPs during tooth development. This speculation is further supported by our observation that the ameloblast differentiation did not seem to be significantly altered in the dcKO mice.

The decreased p-Smad 1/5/8 level and ratio of phosphorylated to total Smad 1/5/8 indicated that BMP2 and BMP4 activated BMP/Smad4 signaling during amelogenesis. On the other hand, the unaltered p-Erk level suggested that BMP/Erk signaling were not activated by BMP2 and BMP4, and contributed few to the MMP20 and KLK4 expression. However, the dramatically reduced pan-Erk in the dcKO enamel organs implicated that signal molecules other than BMPs were responsible for Erk up-regulation in amelogenesis. To clarify if BMP/Smad4 signaling directly regulates the expression of MMP20 and KLK4, and if there is a balance between the canonical and non-canonical BMP signaling during normal amelogenesis, are our aims in the future studies.

In summary, our data in the present study demonstrated that epithelial *Bmp2* and *Bmp4* play critical roles in amelogenesis by regulating the expression of the proteinases MMP20 and KLK4. Our future studies will focus on how the BMP signaling pathways control the maturation of enamel by regulating the expression of these two proteinases.

## Methods

All the experimental methods were approved by the research committee at Texas A&M University Baylor College of Dentistry. All the experiments have been carried out in accordance with the guidelines from the research committee at Texas A&M University Baylor College of Dentistry.

### Generation of *K14-cre;Bmp2* ^*f*/*f*^ *;Bmp4* ^*f*/*f*^(double conditional knockout, dcKO) mice

All animal procedures were approved by the Institutional Animal Care and Use Committee (IACUC) of Texas A&M University Baylor College of Dentistry. *K14-cre* transgenic, *Bmp2*-floxed and *Bmp4*-floxed mice were purchased from Jackson Laboratory^25^,^26^,^27^. By crossbreeding the *Bmp2*^*f*/*f*^*;Bmp4*^*f*/*f*^mice with *K14-cre;Bmp2*^*f***/+**^*;Bmp4*^*f*/+^ mice, we generated *K14-cre;Bmp2*^*f/f*^*;Bmp4*^*f/f*^mice, which we will refer to as*“K14*-*Cre*-mediated double conditional knockout” (dcKO) mice. Tail biopsies were analyzed by polymerase chain reaction (PCR) genotyping with primers recommended by Jackson Laboratory. These primers are specific for the *Bmp2—*floxed allele or *Bmp4-*floxed allele (*Bmp2* forward primer: 5′–TTGGCAAACAGATGCAAGAG-3′, *Bmp2* reverse primer: 5′-TGGACCACACAGAGTCAAGG-3′; *Bmp4* forward primer: 5′-GAGCTAAGTTTTGCTGGTTTGC-3′, *Bmp4* reverse primer: 5′-GCCCATGAGCTTTTCTGAGA-3′). The *Bmp2*^*f*/*f*^*;Bmp4*^*f*/*f*^mice from the same litters as the dcKO mice created during the crossbreeding regime were used as normal controls. Utilizing the *Bmp2*^*f*/*f*^*;Bmp4*^*f*/*f*^littermates of dcKO mice as normal controls not only reduced the number of mice needed but also prevented potential variances that might result from comparing mice out of different litters.

### Plain X-ray Radiography and Micro-computed Tomography (Micro-CT)

The 3-week-old mandibles dissected from *Bmp2*^*f*/*f*^*;Bmp4*^*f*/*f*^(normal), *K14-cre;Bmp2*^*f*/+^*;Bmp4*^*f*/+^*, K14-cre;Bmp2*^*f*/*f*^*;Bmp4*^*f*/+^ and *K14-cre;Bmp2*^*f*/+^*;Bmp4*^*f*/*f*^and *K14-cre;Bmp2*^*f*/*f*^*;Bmp4*^*f*/*f*^(dcKO) mice were fixed for 48 h in 4% paraformaldehyde and stored in 70% ethanol at 4 °C. These jaws were then analyzed by plain X-ray radiography (Faxitron MX-20, FaxitronBioptics, AZ, USA). Then the mandibles from the normal and dcKO mice (4 mice per group) were examined by micro-CT (μCT35, Scanco Medical, Brüttisellen, Switzerland) using a high-resolution scan (3.5 μm slice increment) for morphological observations. For mineral density and enamel thickness measurements, the enamel area of 200 slices centered on the cut-through of the mesial root in the first molar (n = 4).

### Backscattered Scanning Electron Microscopy (SEM)

For the SEM analyses, the mandibles from 4-week-old normal and dcKO mice were fixed with 4% paraformaldehyde and then dehydrated in ascending concentrations of ethanol and embedded in methylmethacrylate (MMA, Buehler, Lake Bluff, IL, USA). The frontal section at the first molar level was mounted, carbon-coated, and examined using a FEI/Philips XL30 SEM system (JSM-6010LA, JEOL, Tokyo, Japan).

### Preparation of Decalcified Sections and Hematoxylin and Eosin (H&E) Staining

The mandibles from postnatal 6-, 10-, 13- and 17-day mice were dissected and fixed in 4% paraformaldehyde in 0.1% diethylpyrocarbonate (DEPC)-treated phosphate-buffered saline (PBS) solution and then decalcified in 0.1% DEPC-treated 15% ethylenediaminetetracetic acid (EDTA) solution at 4 °C. The tissues were processed for paraffin embedding, and 5-μm serial sections were prepared for hematoxylin and eosin (H&E) staining, *in situ* hybridization staining (ISH), and Immunohistochemistry (IHC) staining. H&E staining was performed to examine the enamel histological appearance of the lower first molars from normal and dcKO mice. ISH and IHC were done to analyze the difference in the transcription and proteins distribution of these amelogenesis proteins in enamel and ameloblasts from normal and dcKO mice.

### *In Situ* Hybridization (ISH)

The RNA probes for ameloblastin (AMBN), amelogenin (AMEL), amelotin (AMTN), enamelin (ENAM), odontogenic ameloblast-associatedprotein (ODAM), kallikrein-related peptidase 4 (KLK4) and matrix metalloproteinase 20 (MMP20) were obtained by PCR with mouse incisor cDNA as a template and were synthesized as we described previously^28^. DIG-labeled RNA probes were detected by an enzyme-linked immunoassay with a specific anti-DIG-AP antibody conjugate (Roche, Indianapolis,IN, USA) and an improved substrate (VectorLaboratories, Burlingame, CA, USA), with purple indicating positive signals. Nuclear fast red was used for counterstaining. A detailed description of the protocols of ISH can be found in our previous reports^28^.

### Immunohistochemistry (IHC)

The IHC was performed using an ABC kit and a DAB kit (Vector Laboratories, Burlingame, CA, USA). Briefly, paraffin sections were treated with 3% H_2_O_2_ to block endogenous peroxidase activity followed by heat-induced antigen retrieval incitrate buffer. Then, sections were incubated with 3% bovine serum albumin and 10% normal goat or rabbit serum to avoid nonspecific immunoreactions. Sections were then labeled with primary antibody overnight at 4 °C. Next, sections were incubated with biotinylated secondary antibodies at room temperature for 1 h. Finally, sections were treated with ABC kit (Vector), and immunopositive loci were detected using 3,3′-diaminobenzidine tetrahydrochloride (DAB) solution. Sections were counterstained with methyl green solution. Rabbit polyclonal antibodies against ENAM, AMEL, AMBN, p-Smad 1/5/8, p-JUNK and p-ERK, and goat polyclonal antibodies against ODAM and MMP20 AMTN and KLK4 from Santa Cruz (Santa Cruz Biotechnology, Inc., Dallas, TX), and rabbit monoclonal antibody against p-p38 from Abcam (Abcam, Cambridge, MA, USA) were used following the manufacturer’s instructions.

### Coomassie Brilliant Blue and Western blotting Analysis

Coomassie Brilliant Blue and Western blotting were performed to evaluate the enamel matrix proteins. Total protein was extracted from the enamel organ and enamel matrix of postnatal five day (P5) 1^st^ molar by using ice-cold RIPA lysis buffer system (Santa Cruz Biotechnology, Inc., Dallas, TX) including Protease Inhibitor Cocktail, PMSF in DMSO and sodium orthovanadate in water. The collected samples were homogenized manually with a pestle for 10 s, after centrifugation the supernatant was collected and the protein concentration of the supernatant was measured using the BicinchoninicAcid (BCA) assay (Pierce Biotechnology). Twenty microgram of sample from mouse molar enamel was loaded onto 10% gels for Coomassie Brilliant Blue staining, which was used to profile enamel proteins. AMBN and its cleavage products in the enamel were examined by Western blotting analyses; in these experiments 10 μg of total protein from either of the two groups was loaded in SDS-PAGE and transferred onto Polyvinylidene difluoride (PVDF) membrane (BIO-RAD). The protein-containing membrane was incubated with rabbit-derived anti-AMBN antibody (sc-50534, Santa Cruz Biotechnology, Dallas, TX) at 4 °C for overnight. The goat-derived anti-rabbit IgG conjugated with horseradish peroxidase (sc-2030, Santa Cruz Biotechnology, Dallas, TX; 1: 2000) was used as the secondary antibody. The immunoreactive bands were detected with an Enhanced Chemiluminescence (ECL) detection system (Pierce Biotechnology), according to the manufacturer’s instructions. Chemiluminescent bands were imaged using a CL-XPosure film (Pierce Biotechnology Inc., Rockford, Ill., USA).

Rabbit polyclonal antibodies against p-Samd 1/5/8 (sc-12353-R), total Smad 1/5/8 (pan-Smad 1/5/8; sc-6037-R), p-Erk (sc-23759-R) and total Erk (pan-Erk; sc-94) were purchased from Santa Cruz Biotech Inc. to detected the level of BMP signaling in the P9 enamel organs from normal and dcKO. Mouse monoclonal antibody against β-actin (Sigma-Aldrich, A1978) was used as the inner control. The protein extract and the SDS-PAGE running were performed as above described. The anti-rabbit IgG (Sigma-Aldrich, A9919) and anti-mouse IgG (Sigma-Aldrich, A-2429) conjugated with Alkaline Phosphatase were used as secondary antibody. The CDP-star chemiluminescent was used to deteted the immunostained bands (Sigma-Aldrich,C0712). Quantitative analyses for Western bands were performed by ImageJ.

### Statistical Analysis

Statistical evaluations of the data were conducted by Student’s t test to validate the differences of mineral density and enamel thickness between the normal and dcKO group. *P* < 0.05 was considered statistically significant. The data were presented as mean ± SD.

## Additional Information

**How to cite this article**: Xie, X. *et al.* Abrogation of epithelial BMP2 and BMP4 causes Amelogenesis Imperfecta by reducing MMP20 and KLK4 expression. *Sci. Rep.* **6**, 25364; doi: 10.1038/srep25364 (2016).
